# Trends in full-time working in general practice: a repeated cross-sectional study

**DOI:** 10.3399/BJGP.2023.0432

**Published:** 2024-08-06

**Authors:** Joseph Hutchinson, Jon Gibson, Evangelos Kontopantelis, Kath Checkland, Sharon Spooner, Rosa Parisi, Matt Sutton

**Affiliations:** Centre for Primary Care and Health Services Research;; Centre for Primary Care and Health Services Research;; Division of Informatics, Imaging and Data Science;; Centre for Primary Care and Health Services Research;; Centre for Primary Care and Health Services Research;; Division of Informatics, Imaging and Data Science;; Centre for Primary Care and Health Services Research, University of Manchester, Manchester.

**Keywords:** primary health care, general practice, workforce, workload, contracts

## Abstract

**Background:**

There is little evidence and no agreement on what constitutes full-time working for GPs. This is essential for workforce planning, resource allocation, and accurately describing GP activity.

**Aim:**

To clarify the definition of full-time working for GPs, how this has changed over time, and whether these changes are explained by GP demographics.

**Design and setting:**

Data were obtained from repeated cross-sectional national surveys for GPs, which were conducted between 2010 and 2021.

**Method:**

A comparison was undertaken of three measures of working time commitments (hours and sessions per week and hours per session) plus a measure of workload intensity across survey years. Multiple regression was used to adjust the changes over time for age, sex, ethnicity, contract type, area deprivation, and rurality. Unadjusted hours and sessions per week were compared with definitions of full-time working.

**Results:**

Average hours and sessions per week reduced from 40.5 (95% confidence interval [CI] = 38.5 to 42.5) to 38.0 (95% CI = 36.3 to 39.6) and 7.3 (95% CI = 7.2 to 7.3) to 6.2 (95% CI = 6.2 to 6.3) between 2010 and 2021, respectively. In 2021, 54.6% of GPs worked at least 37.5 hours per week and 9.5% worked at least nine sessions. Hours per session increased from 5.7 (95% CI = 5.7 to 5.7) to 6.2 (95% CI = 6.2 to 6.3) between 2010 and 2021. Partners worked more hours, sessions, and hours per session. Adjustments expanded the increase in hours per session from 0.54 to 0.61.

**Conclusion:**

At the current average duration of sessions, six sessions per week aligns with the NHS definition of full-time hours. However, hours per week is a more consistent way to define full-time work for GPs.

## Introduction

General practice in England is under increasing pressure from patient, system, and supply-side issues.^1^ A key factor is the supply of GPs, with a reported decline in full-time practitioners over the past decade, despite the number of doctors training to be a GP increasing.^2^^,^^3^ At the same time, appointment numbers have remained high and by some measures increased, with a gradual increase in total patient numbers.^2^ As a result, patients are reporting difficulties in obtaining appointments with GPs,^4^ while GPs feel under increasing pressure, with a rising incidence of burnout, early retirement, and willingness to leave the profession.^5^^,^^6^

Discussion of these trends often focuses on a significant proportion of GPs working part-time, with press commentary sometimes implying that this contributes to difficulty with access to health care.^7^^–^9 In response, the Royal College of General Practitioners (RCGP) and individual GPs have contended that a ‘part-time’ GP will often work more hours than other full-time employees.^10^^,^^11^ Solving the current crisis in general practice requires a good understanding of these issues. If GPs are working fewer hours than expected, then solutions should focus on ways to increase GPs’ working hours. Alternatively, if ‘part-time’ working is a misnomer, which does not properly describe the amount of time worked by GPs, then the potential for these solutions is limited.

The study used data from a repeated survey of GPs’ working lives to investigate work trends since 2010. The aims were as follows: 1) to provide clarity on whether measurements of ‘full-time equivalent’ (FTE) are meaningful and useful; 2) to analyse how the duration of a session has changed over time; and 3) to analyse whether these changes are explained by changing GP demographics. The results should enable a more informed discussion of the current crisis.

## Method

### Study design

Responder-level data were obtained from repeated nationwide surveys conducted by The University of Manchester in 2010, 2012, 2015, 2017, 2019, and 2021.^12^^–^17

### National GP Worklife Survey

The GP Worklife Survey (GPWLS) randomly samples GPs working in England.^12^^–^17 It contains questions about their demographics, work practices, and views on work and wellbeing. All returned surveys were pooled from 2010–2021. Data were obtained on sessions and hours worked per week, age, contract types (partner, salaried, or locum), sex, ethnicity, perceptions of workload intensity, and the rurality and deprivation of the practice population.

**Table:** How this fits in

General practice is under increasing pressure, which is in part owing to a lack of GPs. There is contention as to the proportion of GPs working full-time. Average hours and sessions worked per week by GPs in England have declined, while average hours per session have increased. More than half (54.6%) of GPs work at least the NHS standard full-time definition of 37.5 hours per week. Average hours worked per session in 2021 was 49.2% greater than the British Medical Association (BMA) standard definition of a session’s duration. The authors recommend removing sessions as a definition of full-time working. However, if full-time work commitment continues to be defined in terms of the number of sessions worked, alignment with the NHS definition of 37.5 hours per week could be achieved by recognising that 6.0 sessions per week of 6.2 hours constitutes full-time work.

The specific questions used were as follows:

- How many sessions do you work in a typical week? (Free text);
- How many hours do you spend, on average, per week, doing NHS GP-related work? Please include ALL clinical and non-clinical NHS work (Free text);
- What is your age? (Free text);
- Which of the following types of contract do you hold? (Tick box; partner/principal, salaried [including assistant, retainer], locum);
- How would you classify your ethnic background? (Tick box);
- Several measures of work intensity, discussed in the intensity of work section; and.
- Responders could select more than one employment type (partner, salaried, and locum). Responders were classified by partner or not-partner.

The GPWLS consists of cross-sectional and longitudinal elements, whereby some GPs are sampled from the previous survey year. Including longitudinal data would have risked introducing bias, so only the cross-sectional responders were used.

Reported values that were implausible were sequentially excluded, with cut-offs agreed in the research team, including the following: >21 or <1 session per week (*n* = 9 and *n* = 1, respectively); <4 or > 87.5 hours per week (*n* = 54 and *n* = 18, respectively); aged <28 years or >80 years (*n* = 7 and *n* = 4, respectively); or >10 or <2 hours per session (*n* = 134 and *n* = 160, respectively). These were set to missing and multiple imputed.

The GPWLS sample is slightly over-represented by partner, female, and 45–59-year-old GPs (Supplementary Table S1). To account for this, the 2014 GP headcount from NHS Digital was gathered (NHS Digital has now merged with NHS England). It was chosen as it is the closest data release to the midpoint.^18^ Retired GPs and GP registrars were removed, with the remaining GPs stratified by age, sex, and contract type to provide the marginal proportions in Supplementary Table S1. The GPWLS data were then ranked to create a matching weighting vector.

### Income deprivation

Income deprivation score is a measure of the proportion of the local population receiving income-related benefits, released in the 2010, 2015, and 2019 index of multiple deprivation. The data were linked to GPs using the distribution of their practice’s registered patients by lower-layer super output areas. The 2010 and 2012 surveys were linked to 2010 income deprivation scores, while the 2015 and 2017 surveys were linked to 2015 and 2019, and 2021 surveys were linked to 2019.

### Rurality

The rurality of the practice was obtained from the NHS Payments to General Practice dataset,^19^ which defines a practice as either rural or urban depending on the practice postcode.

### Intensity of work

The intensity of work potentially influences working patterns. Factors indicating the intensity of work were obtained from the GPWLS if the question was constant in all survey years. This included increased demands from patients, dealing with problem patients, paperwork, having insufficient time to do the job, dealing with earlier discharges from hospital, unrealistically high expectations of role by others, and interruptions by emergency calls during surgery.

Responses were collected on Likert scales with the GP asked to rate the factors according to how much pressure they experience in their job. They provided a score of 1–5, corresponding to no pressure, slight pressure, moderate pressure, considerable pressure, or high pressure, respectively. To assess whether these variables were measuring the same concept, Cronbach’s alpha was calculated. This equalled 0.84 (95% confidence interval [CI] = 0.83 to 0.85) indicating good internal consistency. Therefore, the mean score across these seven measures was used.

### Missing data

In total, 1140 out of 7340 (15.5%) responders had at least one missing variable, comprising 1.5% of total data-points. Hours per session had the greatest proportion missing (10.9%). Responders with a missing variable varied by year: 19.2% in 2010, 12.4% in 2012, 18.6% in 2015, 12.7% in 2017, 20.8% in 2019, and 12.9% in 2021. This was handled by multiple imputation using the MICE (Multivariate Imputation by Chained Equations) package in R studio (version 4.2.1).^20^ Fifty imputations and chained equations were used. This followed the principle of fully conditional specification that produces less biased analyses than pairwise deletion.^21^ Sessions per week, hours per week, age, income deprivation, hours per session, and intensity were estimated using predictive mean matching. Partner, sex, ethnicity, and rurality were estimated using logistic regression.

### Definitions of full-time working

NHS England defines full-time working as 37.5 hours per week within their national statistics.^3^ The British Medical Association (BMA) divides a GP’s work into sessions, which it assumes last 4 hours and 10 minutes.^22^ The BMA then defines a full-time GP as working nine sessions, which is often used as a basis for pay and contract negotiations.^22^ Meanwhile, the NHS Health Careers website states that eight sessions is full-time in their role description.^23^ In the general labour market, the UK government uses a standard definition of full-time as working more than 35 hours per week and the Office of National Statistics uses 30 hours per week.^24^^–^25 The survey responses were compared with these benchmarks.

### Statistical analysis

Data were handled as pooled repeated cross-sections. Descriptive statistics of the observed weighted variables were calculated. Proportions of the sampled GPs working at or more than nine sessions, eight sessions, 37.5 hours, 35 hours, and 30 hours were calculated disaggregated by survey year. Multiple linear regressions were then conducted on the multiply imputed weighted datasets, with results pooled to analyse the relationship between the survey year and each of the sessions, hours, hours per session, and intensity of work. The reference year was 2010. To analyse whether these results were owing to changes in age, sex, ethnicity, contract type, practice deprivation, or rurality of the GPs and practice populations, the regressions were then repeated while adjusting for these factors.

The following three sensitivity analyses were conducted: 1) pairwise deletion compared with multiple imputation; 2) including the longitudinal data; and 3) including the implausible data. All analyses were conducted in R studio (version 4.2.1).

## Results

**Figure 1.**
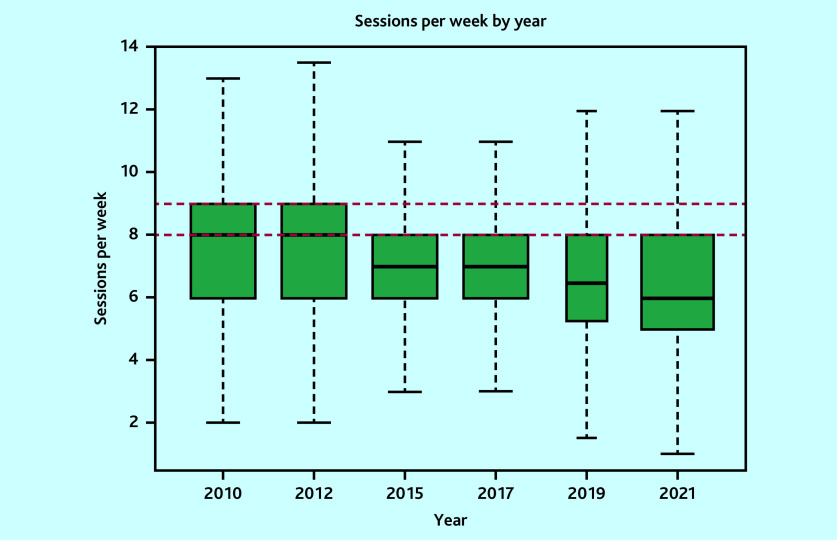
Boxplot of weighted sessions per week by survey year (outlier points removed). Red lines correspond to nine sessions and eight sessions, which are the British Medical Association and the NHS Health Careers website (job description) definitions, respectively.

**Figure 2.**
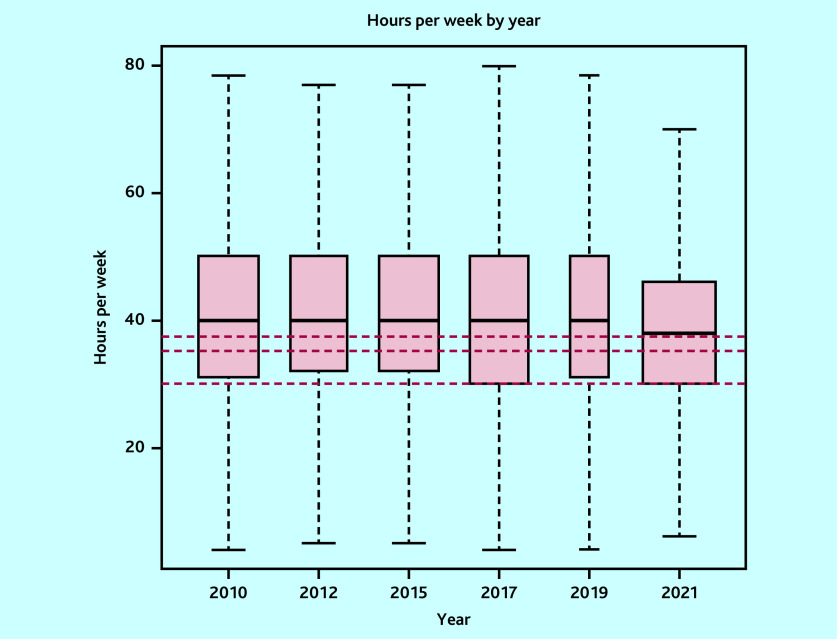
Boxplot of weighted hours per week by survey year (outlier points removed). Red lines correspond to 37.5 hours, 35 hours, and 30 hours, which are the NHS, general UK, and Office for National Statistics definitions, respectively.

**Figure 3.**
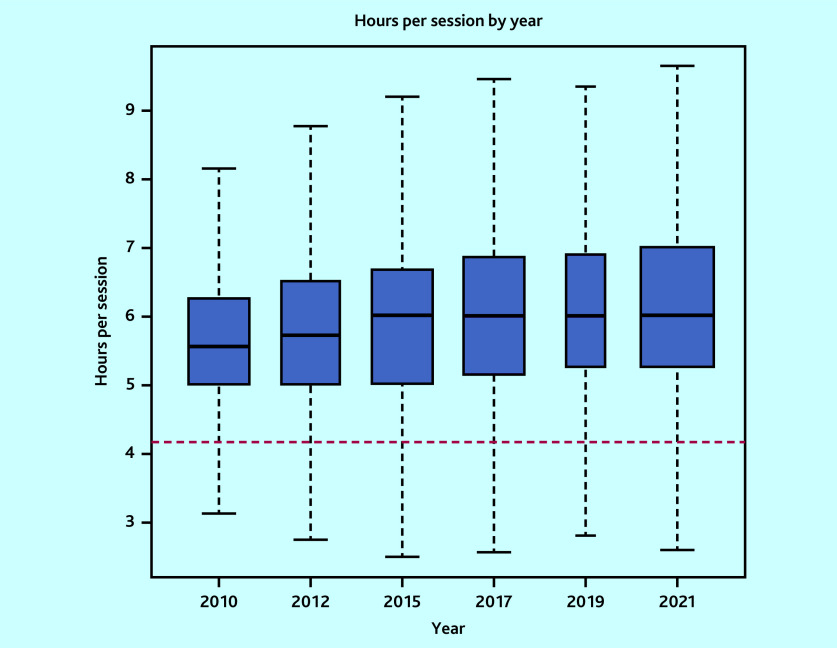
Boxplot of weighted hours per session by survey year (outlier points removed). Red line corresponds to 4 hours 10 minutes, which the British Medical Association uses to define the length of a session.

In total, 7340 surveys were included: 1341 in 2010, 1143 in 2012, 1298 in 2015, 1268 in 2017, 529 in 2019, and 1761 in 2021. The variability in sample size was owing to variations in the target population sizes and response rates from the corresponding surveys.^12^^–^17 Mean sessions per week changed from 7.3 sessions (95% CI = 7.2 to 7.3) to 6.2 sessions (95% CI = 6.2 to 6.3), mean hours per week from 40.5 hours (95% CI = 38.5 to 42.5) to 38.0 hours (95% CI = 36.3 to 39.6), while hours per session from 5.7 hours (95% CI = 5.7 to 5.7) to 6.2 hours (95% CI = 6.2 to 6.3) in 2010 and 2021, respectively (Tables 1 and Figures 1–3). Sessions per week and hours per session showed significant changes compared with 2010. *P* values are <0.05, except for hours per week in the adjusted model, where *P* = 0.06. However, changes in hours per week was less consistent (Supplementary Tables S2–S5).

**Table 1.** Descriptive statistics by survey year for multiple imputed weighted continuous variables

		**2010 (*n* = 1341)**	**2012 (*n* = 1143)**	**2015 (*n* = 1298)**	**2017 (*n* = 1268)**	**2019 (*n* = 529)**	**2021 (*n* = 1761)**
**Sessions per week**	Mean	7.27	7.22	6.90	6.72	6.57	6.24
	95% CI mean	7.22 to 7.33	7.16 to 7.28	6.84 to 6.95	6.67 to 6.77	6.49 to 6.65	6.20 to 6.28
	SD	2.05	1.99	1.98	1.95	1.93	1.92

**Hours per week**	Mean	40.50	41.30	40.86	40.23	39.73	37.98
	95% CI mean	38.49 to 42.50	39.08 to 43.52	38.66 to 43.05	38.07 to 42.39	36.19 to 43.27	36.33 to 39.63
	SD	12.82	12.87	13.70	13.50	14.50	12.89

**Hours per session**	Mean	5.67	5.82	6.03	6.08	6.13	6.22
	95% CI mean	5.65 to 5.71	5.79 to 5.85	5.99 to 6.06	6.05 to 6.12	6.08 to 6.19	6.19 to 6.25
	SD	1.28	1.30	1.37	1.39	1.44	1.51

**Age, years**	Mean	46.52	46.08	45.97	47.68	47.50	45.22
	95% CI mean	44.88 to 48.17	44.29 to 47.89	44.25 to 47.70	45.88 to 49.48	44.65 to 50.35	43.75 to 46.70
	SD	9.15	9.34	9.55	9.51	9.76	9.68

**Income deprivation**	Mean	0.14	0.13	0.14	0.15	0.12	0.12
	95% CI mean	0.14 to 0.14	0.13 to 0.13	0.14 to 0.14	0.15 to 0.15	0.12 to 0.12	0.12 to 0.12
	SD	0.07	0.07	0.07	0.07	0.06	0.06

**Intensity**	Mean	3.45	3.71	4.00	3.98	3.85	3.79
	95% CI mean	3.44 to 3.46	3.70 to 3.72	3.99 to 4.01	3.97 to 3.99	3.82 to 3.87	3.78 to 3.80
	SD	0.71	0.68	0.64	0.67	0.77	0.72

The proportion of GPs working at, or more than the FTE definitions are detailed in Table 3. These proportions have decreased over time across all definitions. However, in 2021, 54.6% of GPs worked at or more than the NHS definition of full-time working (37.5 hours). Only 9.5% of GPs worked nine sessions, used by the BMA to define full-time working. However, the mean hours per session of 6.2 hours in 2021 is 49.2% more than the BMA definition of full-time working.

**Table 2.** Descriptive statistics by survey year for multiple imputed weighted categorical variables

		**2010 (*n* = 1341)**	**2012 (*n* = 1143)**	**2015 (*n* = 1298)**	**2017 (*n* = 1268)**	**2019 (*n* = 529)**	**2021 (*n* = 1761)**
**Contract type^a^**	Partner, *n* (%)	1138 (84.9)	963 (84.3)	1087 (83.7)	1090 (86.0)	395 (74.6)	1255 (71.3)

**Sex^a^**	Male, *n* (%)	588 (43.8)	620 (54.3)	655 (50.5)	652 (51.5)	272 (51.5)	743 (42.2)
	Female, *n* (%)	753 (56.1)	523 (45.7)	643 (49.5)	616 (48.5)	257 (48.5)	1018 (57.8)

**Ethnicity^a^**	White, *n* (%)	1135 (84.7)	958 (83.8)	1031 (79.4)	986 (77.8)	418 (79.0)	1433 (81.4)
	Non-White, *n* (%)	206 (15.3)	185 (16.2)	268 (20.6)	282 (22.2)	111 (21.0)	328 (18.6)

**Rurality^b^**	Urban, *n* (%)	1102 (82.2)	901 (78.8)	1030 (79.4)	1078 (85.0)	416 (78.6)	1450 (82.3)
	Rural, *n* (%)	239 (17.8)	242 (21.2)	268 (20.6)	190 (15.0)	113 (21.4)	311 (17.7)

a *Contract type, sex, and ethnicity refer to how the GP self-identifies, with contract type being either a partner or not.*

b *Rurality refers to the binary geographic coding of their practice location, either rural or urban.*

**Table 3.** Proportions of GPs working at least full-time, using different definitions by survey year

**Organisation setting the definition**	**General purpose of definition**	**FTE definition (per week)**	**2010 %**	**2012 %**	**2015 %**	**2017 %**	**2019 %**	**2021 %**
British Medical Association	Pay and contracts	9 sessions	32.6	28.0	20.0	16.3	14.3	9.5
NHS Health Careers website	Job description	8 sessions	57.9	52.8	45.3	40.2	38.5	29.4
NHS Digital	NHS workforce statistics	37.5 hours	66.7	66.3	63.6	60.1	58.0	54.6
General UK definition (UK government)	General definition	35 hours	73.9	72.9	71.8	69.7	68.5	65.3
Office for National Statistics	National statistics	30 hours	84.1	85.3	84.3	82.4	81.5	81.5

*FTE = full-time equivalent.*

There has been a significant 0.38 (95% CI = 0.33 to 0.43) increase in GP reported work intensity in 2021 compared with 2010 (*P* <0.05), representing a 11.0% increase. However, there has been a steady decline since 2015 indicating mixed trends (Supplementary Table S5 and Figure S1).

The results of the multiple regression models, when adjusting for the potentially confounding variables, are detailed in Supplementary Tables S2–S5. Results of the impact of survey year on sessions per week, hours per week, hours per session, and intensity remained consistent with the unadjusted trends, except for changes in hours per week in 2021, which lost magnitude and significance. Adjustments expanded the increase in hours per session from 0.54 to 0.61 hours. GPs worked slightly fewer (−0.07 [95% CI = −0.12 to −0.02]) hours per session per 10% increase in deprivation, with no significant difference in work intensity or sessions or hours per week.

Compared with non-partners, partners consistently worked significantly more hours and sessions and more hours per session and reported greater work intensity (*P* <0.05). Meanwhile, Black and Minority Ethnic (BAME) and male doctors worked significantly more hours and sessions but fewer hours per session (*P* <0.05).

The results of the sensitivity analyses did not change the main findings (Supplementary Tables S6–S9), with no difference from removing the implausible values above.

## Discussion

### Summary

The percentage of GPs working NHS full-time hours per week was 54.6% in 2021, much greater than the 25% figure recently reported.^8^^,^^9^ Moreover, the average number of hours per session in 2021 was 49.2% more than the BMA’s official definition.^22^ The number of hours worked per session by GPs has increased since 2010, with the number of sessions and hours worked decreasing. Similarly, intensity of work is significantly greater in 2021 than in 2010.

Partners worked more hours, sessions, and hours per session than non-partners. Male and BAME GPs worked more hours and sessions per week, but fewer hours per session.

### Strengths and limitations

GP-level survey data were used, which was supplemented with practice deprivation and rurality. This enabled the analysis of how GP work practices have changed over time, while controlling for key potential confounding variables.

The results rely on survey data accurately reflecting GP work practices. The GPWLS involves several processes to ensure a reasonable response rate, making the validity of the findings reassured.^26^ The details of these processes can be found in the individual reports.^12^^–^17 However, the response rates have declined in recent years, which the survey team have responded to by making methodological changes (detailed in Supplementary Table S10). This decline is consistent with UK and worldwide trends.^27^ The independent reporting of hours and sessions is a major advantage for this study. This is because other data sources presuppose a FTE definition.

Responders were asked about all NHS GP-related work, but their interpretation might have been variable.

Multiple imputation was conducted to address the issue of missing data. The sensitivity analysis showed minimal differences between the multiple imputation and pairwise deletion approaches, meaning the impact of this missing data should be minimal.

### Comparison with existing literature

Congruent with previous publications, a reduction in the average number of sessions and hours worked by GPs has been found.^2^^,^^3^ However, the study identified that the number of hours worked in each session has increased over time. Interestingly, the reduction in hours worked per week lost significance when adjusting for changes in the composition of the GP workforce, particularly partners. There is variation in the reported proportion of GPs working full-time,^2^^,^^3^^,^^7^^–^10 which this study clarifies. The current definition of full-time working given by the NHS Health Careers website and the BMA is that a full-time GP will work eight or nine sessions.^22^^,^^23^ The present study suggests this definition is inappropriate when considered according to working hours. The standard NHS definition of full-time hours is 37.5 hours per week. By this measure, 54.6% of GPs work full-time hours, but only 29.4% and 9.5% of GPs work at least eight or nine sessions, respectively. This suggests that current estimates of how many GPs work full-time are likely underestimates. If sessions continue to be used to define full-time equivalency, then a more appropriate definition would be 6.0 sessions a week, which corresponds to the 37.5 hours used for other NHS workers.^3^ However, a more consistent approach would use hours per week.

Interestingly, the GPs age had minimal and inverse impact on hours and sessions per week. This indicates that younger GPs work more, despite a low desire to work full-time by trainee GPs.^28^ Further, average GP work intensity remains moderate to considerable and is a priority for future research.

### Implications for research and practice

This paper has emphasised the importance of understanding the general practice workforce before making policy decisions.

The amount of work performed per session is becoming increasingly underestimated, which may lead to health planners underestimating the associated resources required for commissioning and employment changes. As such, the authors recommend removing sessions as a definition of full-time working, instead using 37.5 hours per week to align with the wider NHS. This would equate to six sessions. This may need a readjustment of salaries accordingly. The accuracy of practice reports to NHS England is also vital in this process.

Fewer GPs are working part-time than expected, so policies attempting to improve access to general practice by increasing hours worked may not be effective. Accurate measures of full-time GPs are required for long-term workforce planning, with further research required to understand these trends.

Salaries for GPs are primarily calculated by the number of sessions they work, as opposed to the number of hours. As such, the steady increase in the numbers of hours worked per session may lead to a relative decline in the GP salary per hour, particularly for salaried and partner GPs.^29^ Further, hours per session varies by GP sex, age, and ethnicity, which may be a source of salary inequality in the profession, such as for female GPs.^30^

Partners worked more sessions and hours per week, as well as more hours per session. It is not known whether GPs who wish to work longer hours are more likely to become partners. However, it is important to be cognisant of this increased work if changing the partnership model.^31^

## Data

Not available.
